# Supplementary material for: INO-4800 DNA vaccine induces neutralizing antibodies and T cell activity against global SARS-CoV-2 variants
Source: NPJ Vaccines. 2021 Oct 14;6:121. doi: 10.1038/s41541-021-00384-7 (PMC8516974; doi:10.1038/s41541-021-00384-7)
Supplement: Supplementary file 1 — Supplementary Information [file 41541_2021_384_MOESM1_ESM.pdf]

Supplementary Table 1. ID50 titers of thirteen INO-4800 vaccinee sera against Wuhan and SARS-CoV-2 variants pseudotyped viruses.

		SARS-CoV-2 pseudo neutralization ID50			
Sample ID	Dose (mg)*	Wuhan	B.1.117	B.1.351	P.1
P1	1	71	46	41	66
P2	1	599	545	243	738
P3	1	407	180	26	382
P4	2	319	188	48	282
P5	2	978	887	310	1292
P6	2	368	151	52	453
P7	1	372	185	33	406
P8	2	411	215	64	339
P9	2	251	167	60	297
P10	2	2550	493	142	2079
P11	2	1766	610	255	2087
P12	2	84	84	37	25
P13	0.5	187	89	53	190
n		13	13	13	13
Mean		643.3	295.4	105	664.3
SD		729	255	98.87	706.7
Range min		70.7	46.5	25.6	25.3
Range max		2550	886.7	309.8	2087

*The dose corresponds to the amount in mg of INO-4800 delivered per immunization period (n=1, 0.5 mg vaccine dose; n = 4, 1.0 mg; n = 8, 2.0 mg).

Supplementary Table 2. Descriptive analysis and geometric mean titers (GMT) of neutralizing ID50 values for Wuhan, B.1.1.7, B.1.351 and P.1 variants.

	Wuhan	B.1.1.7	B.1.351	P.1
Number of subjects	13	13	13	13
Minimum	70.65	46.45	25.95	25.34
Maximum	2550	886.7	309.8	2087
Range	2479	840.3	283.9	2062
Mean	643.2	294.6	104.9	664.4
Std. Deviation	729	255.5	98.84	706.7
Std. Error of Mean	202.2	70.85	27.41	196
Lower 95% CI of mean	202.7	140.2	45.19	237.3
Upper 95% CI of mean	1084	449	164.6	1091
Geometric mean	393	210.4	73.63	372.3
Geometric SD factor	2.822	2.379	2.307	3.493
Lower 95% CI of geo. mean	209.9	124.6	44.42	174.8
Upper 95% CI of geo. mean	735.6	355.2	122	792.8

a

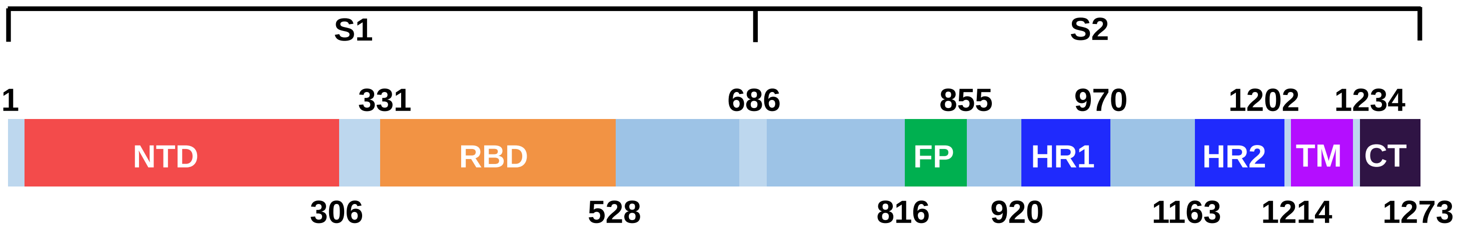

b

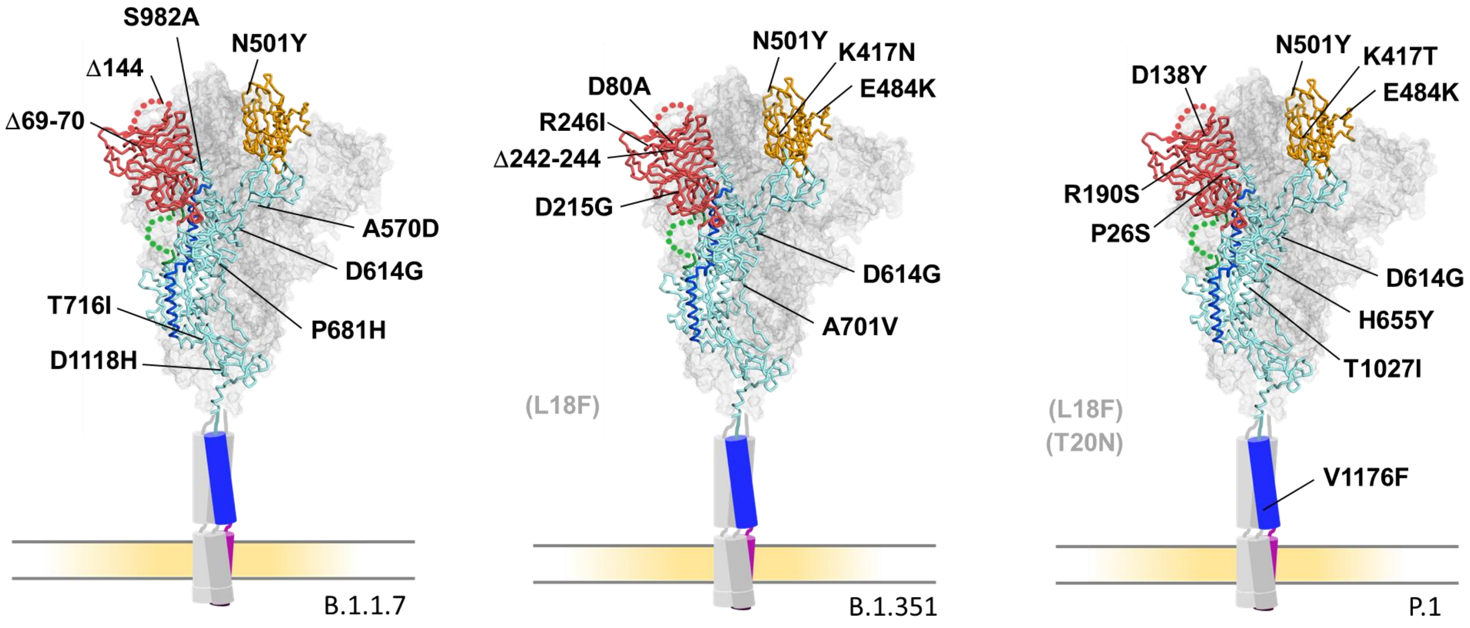

Supplementary Figure 1. Schematic diagram and molecular modeling of SARS-CoV-2 spike protein. A) Spike protein diagram with major features labeled: N-terminal domain (NTD, red), receptor binding domain (RBD, orange), fusion peptide (FP, green), heptad repeats 1 and 2 (HR1 and HR2, blue), transmembrane region (TM, violet), C-terminal domain (CT, dark purple). B) Molecular models of spike protein with mutations indicated for B.1.1.7, B.1.351, and P.1 variants. Trimer model is depicted with one subunit as a Ca trace and colored identically to the diagram in (A) with the two subunits outlined for clarity. Large loops not modeled are indicated by dashed lines and the stalk and membrane-spanning portion of the molecule are indicated with cylinders.
