# Supplementary material for: INO-4800 DNA vaccine induces neutralizing antibodies and T cell activity against global SARS-CoV-2 variants
Source: NPJ Vaccines. 2021 Oct 14;6:121. doi: 10.1038/s41541-021-00384-7 (PMC8516974; doi:10.1038/s41541-021-00384-7)
Supplement: Supplementary file 2 — Reporting Summary [file 41541_2021_384_MOESM2_ESM.pdf]

nature research

Corresponding author(s): Kate Broderick

Last updated by author(s): 06/04/2021

Reporting Summary

Nature Research wishes to improve the reproducibility of the work that we publish. This form provides structure for consistency and transparency in reporting. For further information on Nature Research policies, see our Editorial Policies and the Editorial Policy Checklist.

Statistics

For all statistical analyses, confirm that the following items are present in the figure legend, table legend, main text, or Methods section.

n/a	Confirmed	
☐	☒	The exact sample size (*n*) for each experimental group/condition, given as a discrete number and unit of measurement
☐	☒	A statement on whether measurements were taken from distinct samples or whether the same sample was measured repeatedly
☐	☒	The statistical test(s) used AND whether they are one- or two-sided<br>*Only common tests should be described solely by name; describe more complex techniques in the Methods section.*
☒	☐	A description of all covariates tested
☒	☐	A description of any assumptions or corrections, such as tests of normality and adjustment for multiple comparisons
☐	☒	A full description of the statistical parameters including central tendency (e.g. means) or other basic estimates (e.g. regression coefficient) AND variation (e.g. standard deviation) or associated estimates of uncertainty (e.g. confidence intervals)
☐	☒	For null hypothesis testing, the test statistic (e.g. *F*, *t*, *r*) with confidence intervals, effect sizes, degrees of freedom and *P* value noted<br>*Give P values as exact values whenever suitable.*
☒	☐	For Bayesian analysis, information on the choice of priors and Markov chain Monte Carlo settings
☒	☐	For hierarchical and complex designs, identification of the appropriate level for tests and full reporting of outcomes
☒	☐	Estimates of effect sizes (e.g. Cohen's *d*, Pearson's *r*), indicating how they were calculated

Our web collection on statistics for biologists contains articles on many of the points above.

Software and code

Policy information about availability of computer code

Data collection	Gen 5 v. 3.10 ans and CTL ImmunoCapture and Immunospot v. 7.0
Data analysis	All data analyses were done using GraphPad Prism version 8.1.2.

For manuscripts utilizing custom algorithms or software that are central to the research but not yet described in published literature, software must be made available to editors and reviewers. We strongly encourage code deposition in a community repository (e.g. GitHub). See the Nature Research guidelines for submitting code & software for further information.

Data

Policy information about availability of data

All manuscripts must include a data availability statement. This statement should provide the following information, where applicable:

- Accession codes, unique identifiers, or web links for publicly available datasets
- A list of figures that have associated raw data
- A description of any restrictions on data availability

The data that support the findings of this study are available from the corresponding authors upon reasonable request.

Field-specific reporting

Please select the one below that is the best fit for your research. If you are not sure, read the appropriate sections before making your selection.

☒ Life sciences ☐ Behavioural & social sciences ☐ Ecological, evolutionary & environmental sciences

For a reference copy of the document with all sections, see nature.com/documents/nr-reporting-summary-flat.pdf

Life sciences study design

All studies must disclose on these points even when the disclosure is negative.

Sample size	The sample size was determined based on sample availability and on recent studies on the field showing significant differences across groups using similar sample sizes present in this study. Between 13 and 20 sera samples were used for ELISA and pseudo neutralization assays. PBMCs from 10 subjects were used in an IFN gamma ELISpot.
Data exclusions	No data were excluded from the analysis.
Replication	The assays performed in this study correspond to one independent experiment with the mean of technical duplicates or triplicates.
Randomization	Samples were not randomized due to the exploratory nature of the study and chosen based on availability.
Blinding	The investigators were not blinded during sample allocation or data analysis. However, all three assays were performed by independent investigators and data revealed during assembly of the manuscript.

Reporting for specific materials, systems and methods

We require information from authors about some types of materials, experimental systems and methods used in many studies. Here, indicate whether each material, system or method listed is relevant to your study. If you are not sure if a list item applies to your research, read the appropriate section before selecting a response.

Materials & experimental systems

n/a	Involved in the study	
☐	☒	Antibodies
☐	☒	Eukaryotic cell lines
☒	☐	Palaeontology and archaeology
☒	☐	Animals and other organisms
☐	☒	Human research participants
☐	☒	Clinical data
☒	☐	Dual use research of concern

Methods

n/a	Involved in the study	
☒	☐	ChIP-seq
☒	☐	Flow cytometry
☒	☐	MRI-based neuroimaging

Antibodies

Antibodies used	HRP-Anti-Human IgG; Clone G18-145; Isotype: Mouse IgG1, Kappa; BD Biosciences Cat#555788; Lot: 0191085
Validation	Manufacturer website: The G18-145 monoclonal antibody specifically binds to the heavy chain of human immunoglobulin G subclasses: IgG1, IgG2, IgG3 and IgG4. The G18-145 antibody has been reported not to react with the heavy chains of other human immunoglobulin isotypes. Reference: Zola H, Macardle PJ, Flego L, Webster J. The expression of sub-population markers on B cells: a re-evaluation using high-sensitivity fluorescence flow cytometry. Dis Markers. 1991; 9(2):103-118.

Eukaryotic cell lines

Policy information about cell lines

Cell line source(s)	293T (ATCC® CRL-3216™) and ACE2-CHO cells (ACE2 expressing CHO cells - Creative Biolabs)
Authentication	ACE2-CHOs were tested for permissiveness to SARS-CoV-2 pseudovirus before assays were performed. No other authentication was performed.
Mycoplasma contamination	Cells were not tested for mycoplasma contamination.
Commonly misidentified lines (See ICLAC register)	None.

Human research participants

Policy information about studies involving human research participants

Population characteristics	Healthy participants 18+ years of age with no known prior history of COVID-19 symptoms.
Recruitment	Trial participants were invited to enroll at the following three locations: The University of Pennsylvania (Philadelphia, PA), Center for Pharmaceutical Research (Kansas City, MO), and Central Kentucky Research Associates (Lexington, KY).
Ethics oversight	The institutional review board of each clinical site approved the trial. All trial participants provided written informed consent.

Note that full information on the approval of the study protocol must also be provided in the manuscript.

Clinical data

Policy information about clinical studies

All manuscripts should comply with the ICMJE guidelines for publication of clinical research and a completed CONSORT checklist must be included with all submissions.

Clinical trial registration	NCT04336410
Study protocol	The study protocol can be accessed on clinicaltrials.gov
Data collection	Enrollment began in April 2020, and data collection is ongoing.
Outcomes	Primary and secondary outcomes and their preliminary results are published in Tebas P, Yang S, Boyer JD, et al. Safety and immunogenicity of INO-4800 DNA vaccine against SARS-CoV-2: A preliminary report of an open-label, Phase 1 clinical trial. EClinicalMedicine 2021; 31: 100689.
